# Interaction of target distance and movement type on lower limb kinematics in fencing

**DOI:** 10.3389/fspor.2026.1756804

**Published:** 2026-03-23

**Authors:** Kenta Chida, Takayuki Inami, Shota Yamaguchi, Yasumasa Yoshida, Naohiko Kohtake

**Affiliations:** 1 Department of Policy Management, Keio University, Fujisawa, Japan; 2 Institute of Physical Education, Keio University, Yokohama, Japan; 3 Graduate School of System Design Management, Keio University, Yokohama, Japan

**Keywords:** fencing, lunge, lower limb kinematics, hip angle, knee angle, ankle angle

## Abstract

**Introduction:**

Since the distance between opponents constantly changes during fencing bouts, clarifying the relationship between movement type and target distance is important for understanding tactical movement selection.

**Methods:**

Twelve skilled male foil fencers performed lunges under six conditions combining three target distances (Short, Normal, Long) and two movement types (lunge without advance: LWOA; lunge with advance: LWA). Three-dimensional motion capture (500 Hz) was used to measure hip, knee, and ankle joint angles of the front and rear legs and center of mass (CoM) velocity. For each condition, the trial with the maximum horizontal peak CoM velocity was selected for analysis using a two-factor repeated-measures ANOVA.

**Results:**

Peak CoM velocity increased linearly with target distance and was consistently higher in LWA than in LWOA. The velocity difference between movement types was greatest at the Short distance and decreased at the Long distance. Correspondingly, rear-leg joint kinematics increased with target distance and were consistently larger in LWA than in LWOA, with significant interaction effects.

**Discussion:**

These findings suggest that the effectiveness of attack movements in fencing depends on the interaction between target distance and movement type. Fencers should select LWOA or LWA according to tactical distance while emphasizing rear-leg flexion and extension movements during training.

## 1 Introduction

Advances in camera technology now enable quantitative analysis of movement techniques in many competitive sports, which previously relied on empirical observation and tacit knowledge (1). Fencing is no exception: recent research has provided valuable insights by focusing on athletes’ joint kinematics (2–6). In particular, the fencing lunge is a fundamental and crucial attacking movement in offensive situations. The ability to execute an effective lunge directly impacts scoring and is considered a key factor in match outcomes (7, 8). Although a lunge may appear simple, it requires split second decision making, explosive acceleration, and precise lower body motor control (9). For example, a faster lunge that rapidly reduces the interpersonal distance may allow a fencer to execute a valid touch before the opponent completes a defensive action. Thus, analyzing the lunge can benefit performance enhancement and coaching, including improving training methods for developing athletes.

Fencing attack movements can be broadly categorized as two types: lunges without advance (LWOA) and lunges with advance (LWA), which involve adding a forward step to quickly close the distance (4). In recent years, the kinematic characteristics of LWOA and LWA have been analyzed using indicators such as lower limb joint angles, range of motion (ROM), and center of mass (CoM) velocity. Previous studies have shown that as the target distance increases, the peak velocity of the CoM during the lunge and lower limb joint angle variables change significantly, with the magnitude of these changes being greater for the LWA than for the LWOA (4, 6). However, LWA has only been examined under a single distance condition, and the effects of different target distances on its operation remain unclear. In actual bouts, the distance between opponents constantly changes as they advance and retreat. Therefore, movement type (LWOA vs. LWA) and target distance likely interact to influence lunge kinematics. Nevertheless, how target distance and movement type interact to shape the kinematic characteristics of the lunge remains unclear.

The purpose of this study was to examine the effects of three target distances (Short, Normal, Long) on lower limb joint kinematics (particularly front and rear leg joint motion) and CoM velocity for both LWOA and LWA in fencing. We aimed to clarify how target distance and attack movement type interact to influence performance metrics. We hypothesized that peak horizontal CoM velocity and key lower limb joint variables would increase with target distance in both movement types. In addition, we expected that LWA would produce larger absolute values and greater distance-related increases than LWOA.

## 2 Materials and methods

### 2.1 Participants

The study included able-bodied male members of a university fencing team. The sample size was calculated beforehand using G-Power, with a significance level of *α* = 0.05 and statistical power of 0.8 (10). Previous studies on lunge performance have reported relatively large effect sizes for peak CoM velocity and lower limb joint kinematics (4, 6). Therefore, an effect size of 0.40 was adopted for the *a priori* power analysis. According to Cohen's (1988) conventions, an effect size of 0.40 corresponds to a large effect. Based on this assumption, the required minimum sample size was estimated to be 12 participants. The subjects were 12 male fencers specializing in foil (age: 19.5 ± 0.9 years, height: 171.2 ± 5.6 cm, weight: 63.5 ± 5.7 kg, fencing experience: 9.4 ± 3.0 years; all values expressed as mean ± SD), who were competitors in the National Cup. All subjects were right-handed. Prior to the experiment, the purpose and methods were thoroughly explained both in writing and verbally. Subjects signed informed consent forms only after fully understanding the written content. Participants were confirmed to have no musculoskeletal injuries during the six months prior to testing. This study was conducted with the approval of the Ethics Committee of the Graduate School of System Design and Management, Keio University (Approval Number: SDM-2022-E001).

### 2.2 Trial conditions

**Figure 1:**
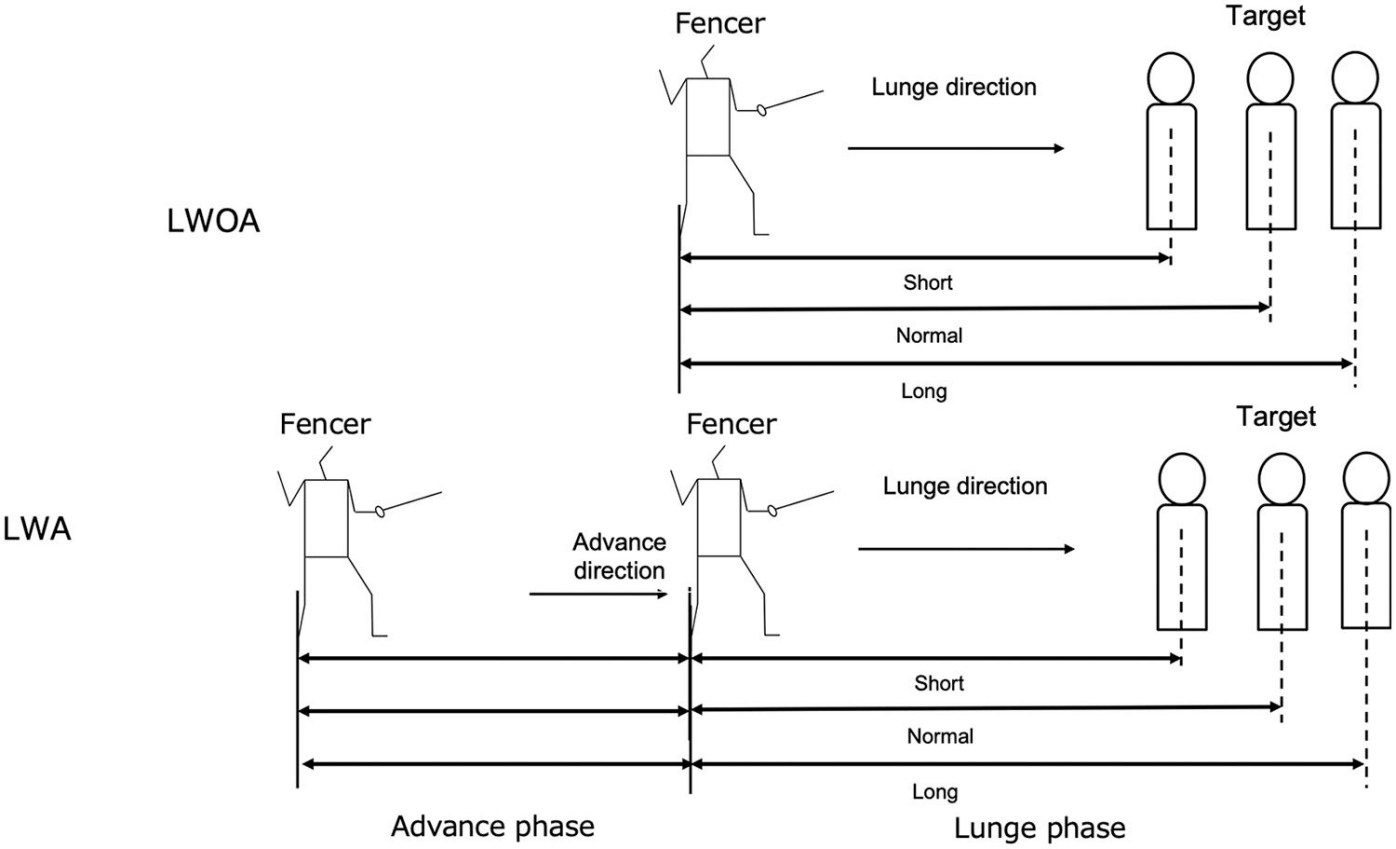
Experimental conditions (short, normal, long distances combined with LWOA or LWA attacks).

Figure 1 shows the experimental conditions. In this study, we set a total of 6 conditions by combining three target distances (Short, Normal, Long) with two movement types (LWOA and LWA). Following prior research (6, 11), Normal distance was defined as 1.5 times the fencer's height, measured from the rear foot toes to the target in the en garde stance. Short distance was defined as Normal distance minus 50 cm (closer to the fencer), and Long distance was Normal plus 30 cm (farther from the fencer) (6). The actual distances were approximately 206.8 ± 8.5 cm for Short, 256.8 ± 8.5 cm for Normal, and 286.8 ± 8.5 cm for Long. These distances were used for both LWOA and LWA conditions. In the LWA conditions, an initial advancing step was performed covering a distance approximately equal to the fencer's shoulder width (distance between the acromion processes); immediately after this advance, the lunge was executed (4). This advance distance definition was based on Chida et al. (2023). The realized advance length was calculated as 45.9 ± 1.9 cm (mean ± SD). All trials were performed toward a target board (30 cm × 30 cm lamé fabric) adjusted to the fencer's chest height so that the foil tip would hit the target surface horizontally.

### 2.3 Procedure

Participants performed a self-directed warm-up (stretching, running, and fencing footwork) for 15 min before testing. Subsequently, to become accustomed to the experimental conditions, they practiced attack movements several times under each of the six conditions. Subsequently, participants assumed the starting position with “En garde.” Participants initiated the lunge from a stationary position at their own discretion and performed three trials per condition (resulting in 18 trials per athlete). To avoid fatigue effects, a 30 s rest period was set between trials. Unsuccessful trials were defined as missing the 30 cm × 30 cm target area, interrupting the movement during the lunge, or showing a clear loss of balance. Ultimately, participants performed a total of 18 trials (3 trials per condition) until successful completion (12). All participants achieved the target distance for each condition, but including failed trials, they performed an average of 3.0 trials for Short, 3.2 trials for Normal, and 3.6 trials for Long in LWOA; and 3.8 trials for Short, 3.6 trials for Normal, and 3.8 trials for Long in LWA. Each participant was instructed to lunge at maximum speed without stopping movement from the start to the touch point in all trials.

### 2.4 Data collection

**Figure 2:**
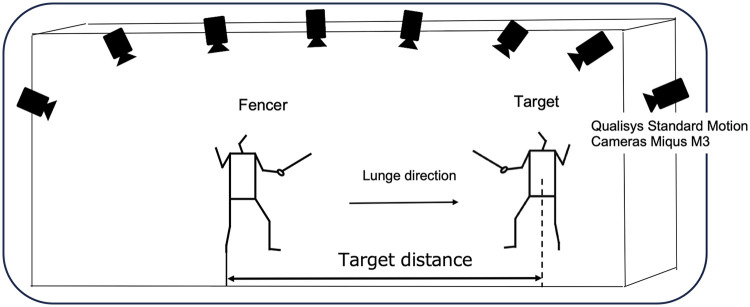
Experimental setup for motion capture.

Figure 2 shows the experimental setup. A three-dimensional motion capture system was used for data collection. Eight high resolution cameras (Qualisys Standard Motion Cameras Miqus M3) were arranged to surround the participant, with a sampling frequency set to 500 Hz. Data captured by the camera was stored in the data station of the three-dimensional motion analysis system using a dedicated cable. In this study, a stationary coordinate system was established with the lunge's forward direction as the *Y*-axis, the direction perpendicular to the *Y*-axis as the *X*-axis, and the vertical direction as the Z-axis. A total of 57 reflective markers (14 mm diameter) were placed on anatomical landmarks and the foil. Full details of marker placement are provided in Supplementary Table S1. For the foil, 7 points were attached to the top of the blade, near the top, middle of the blade, near the guard, guard (guard center, guard right, guard left), totaling 57 points. In this study, the foil No. 5 swords (BF Allstar, Germany; blade length 90 cm) and masks (Allstar, Germany) used by the subjects complied with international standards and were uniform. Participants used their own shoes. Before the trials, participants were positioned at anatomical landmarks, and static trials were collected.

### 2.5 Data analysis

**Figure 3:**
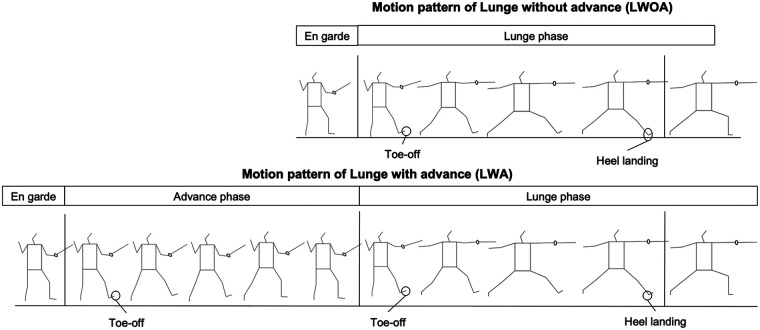
Motion patterns of LWOA (top) and LWA (bottom).

We defined the lunge phase based on prior studies (3, 13). Figure 3 illustrates the motion patterns of the two attack types (LWOA and LWA) analyzed in this study. Participants initiated the movement from an en garde position with both feet on the ground. The start of the lunge was defined as the toe-off of the front foot. The end of the movement was defined as the heel contact of the front foot, following a coordinated forward swing of the front foot in conjunction with the rear foot (3, 14). For each participant and target distance condition, the trial with the highest horizontal CoM velocity among the three successful trials was adopted as the representative value. In addition, previous research has demonstrated significant associations between peak horizontal CoM velocity and lower limb joint angle variables (4), supporting its relevance as a performance-related metric aligned with the kinematic focus of the present study. Time normalization was performed by setting the Start-to-End time as 100%, and the mean and standard deviation of each trial were calculated at 1% intervals.

Marker data were tracked and labeled using Qualisys Track Manager software and exported as C3D files. Three-dimensional marker coordinates were processed in Visual 3D (C-Motion Inc.). All coordinate data were filtered with a 4th-order zero-lag Butterworth low-pass filter (8 Hz cutoff). Local coordinate systems were constructed for each segment (head, thorax, upper arm, forearm, hand, pelvis, thigh, shin, foot). The CoM was calculated using the Visual 3D skeleton model as a reference, and the horizontal plane movement velocity during the participant's lunge was calculated. The pelvis was calculated based on the positions of the ASIS and PSIS markers, while the hip joint center was calculated using the Visual 3D regression equation derived from Bell et al. (15). The knee joint center was calculated as the midpoint between the lateral and medial femoral condyles, and the ankle joint center as the midpoint between the lateral and medial malleoli.

### 2.6 Statistical analysis

Statistical analyses for this study were performed using IBM SPSS Statistics (version 29, IBM Corp., Armonk, NY, USA). Prior to analysis, the normality of all variables was confirmed using the Shapiro–Wilk test. A two-way repeated measures ANOVA was conducted to examine the effects of movement type (LWOA vs. LWA) and target distance (Short, Normal, Long) on the peak horizontal CoM velocity during the lunge. Because the distance factor represented three ordered levels (Short < Normal < Long), the linear trend component was specified *a priori* as the primary contrast to evaluate monotonic distance-dependent changes. When Mauchly's test indicated violation of sphericity, the Greenhouse–Geisser correction was applied. Statistical significance was set at *p* < 0.05. Partial eta-squared (*η*p²) values are reported as effect size measures for the ANOVA results.

## 3 Results

**Figure 4:**
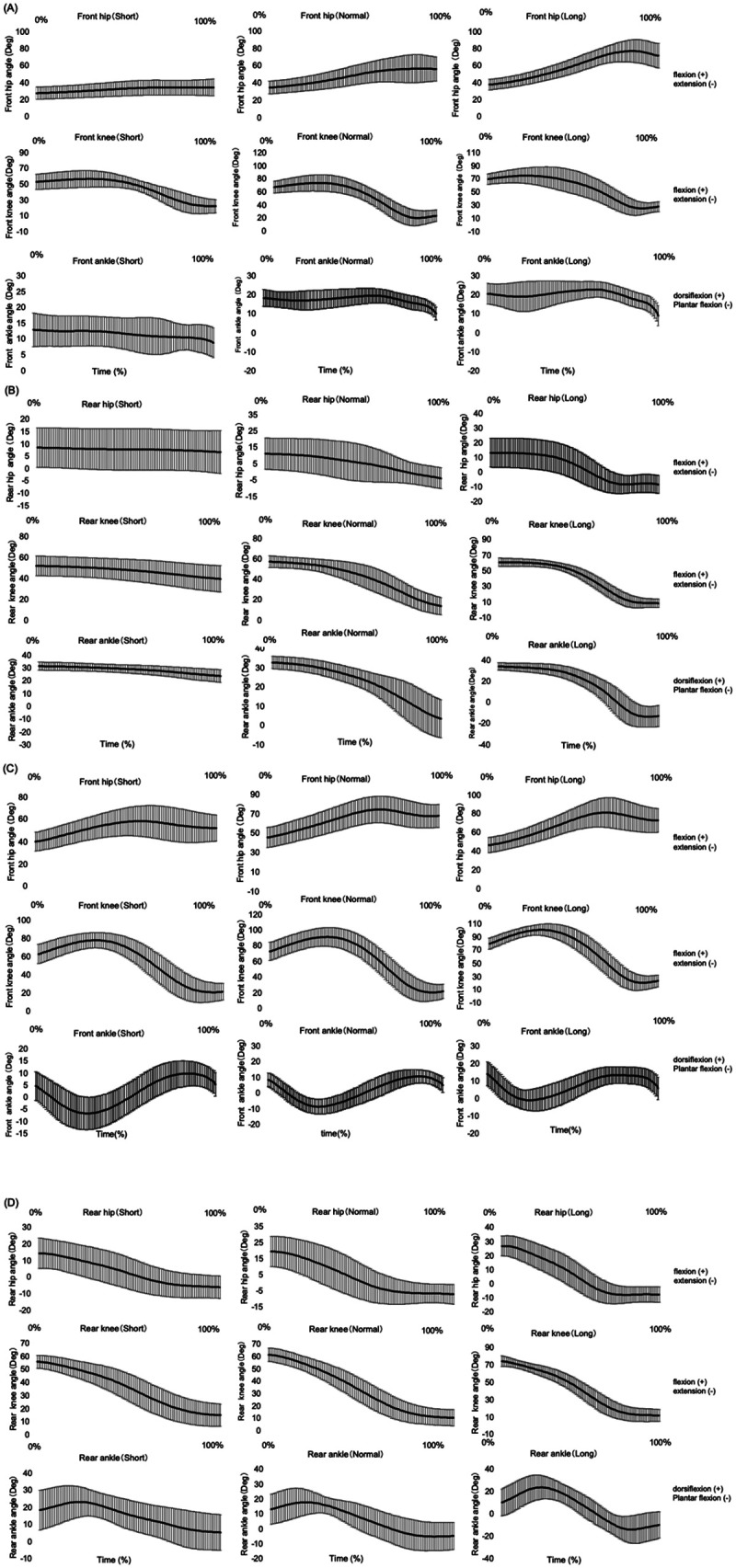
Time-series joint angle patterns for LWOA and LWA lunges. **(A)** Front leg joint angles in LWOA, **(B)** rear leg joint angles in LWOA, **(C)** front leg joint angles in LWA, **(D)** rear leg joint angles in LWA.

Figure 4 displays the time-series patterns of hip, knee, and ankle joint angles changes in the rear and front legs under both LWOA and LWA conditions. Table 1 presents the mean ± SD values for peak CoM velocity during the lunge phase and selected lower limb joint angle variables for each condition. Table 2 summarizes the two-way ANOVA results for the main effects of target distance and movement type and their interaction for each variable. The following results describe these findings in detail.

**Table 1:** Overview of peak CoM velocity and lower limb joint angle variables (hip, knee, ankle) for the rear and front legs (mean ± SD for *N* = 12).

Variable		Target distance condition					
		Short: LWOA	Short:LWA	Normal:LWOA	Normal:LWA	Long:LWOA	long:LWA
CoM	Peak velocity (m/s)	1.02 ± 0.20	1.98 ± 0.19	1.81 ± 0.22	2.42 ± 0.21	2.42 ± 0.24	2.88 ± 0.24
Rear hip	Peak flex (°)	9.81 ± 8.33	15.42 ± 9.16	11.34 ± 10.07	20.19 ± 9.92	13.74 ± 9.04	27.32 ± 7.80
	Peak Ext (°)	4.97 ± 8.69	−6.71 ± 7.38	−4.57 ± 6.98	−8.36 ± 6.62	−9.57 ± 7.01	−9.63 ± 6.16
	ROM (°)	4.84 ± 4.08	22.13 ± 5.60	15.92 ± 6.85	28.54 ± 6.81	23.31 ± 4.92	36.95 ± 4.22
Rear knee	Peak flex (°)	51.90 ± 9.63	56.08 ± 5.10	57.25 ± 5.86	61.40 ± 5.67	60.96 ± 5.25	74.45 ± 6.12
	Peak Ext (°)	38.96 ± 13.11	14.45 ± 9.02	13.17 ± 8.80	8.39 ± 5.43	5.94 ± 6.14	10.36 ± 8.01
	ROM (°)	12.94 ± 10.01	41.64 ± 6.92	44.08 ± 7.82	53.01 ± 8.10	55.01 ± 7.44	64.08 ± 6.48
Rear ankle	Peak DF (°)	31.46 ± 3.23	24.75 ± 8.67	32.48 ± 3.53	19.39 ± 9.20	33.62 ± 3.73	24.03 ± 11.15
	Peak PF (°)	23.47 ± 5.26	2.62 ± 9.99	3.16 ± 10.67	−8.47 ± 9.27	−15.34 ± 10.24	−16.51 ± 10.65
	ROM (°)	7.99 ± 5.88	22.13 ± 10.07	29.32 ± 9.91	27.86 ± 13.54	48.96 ± 9.33	40.54 ± 13.91
Front hip	Peak flex (°)	36.08 ± 10.52	59.48 ± 14.62	59.72 ± 16.37	77.03 ± 12.66	80.43 ± 13.20	83.25 ± 14.50
	Peak Ext (°)	27.21 ± 7.55	39.38 ± 9.28	34.30 ± 7.78	44.94 ± 9.99	37.27 ± 6.65	46.15 ± 8.47
	ROM (°)	8.87 ± 5.28	20.10 ± 11.58	25.42 ± 14.52	32.09 ± 9.07	43.16 ± 14.52	37.10 ± 12.39
Front knee	Peak flex (°)	56.83 ± 10.94	79.41 ± 8.54	74.00 ± 13.13	92.00 ± 12.27	76.42 ± 13.24	101.21 ± 8.57
	Peak Ext (°)	19.99 ± 11.03	18.46 ± 10.83	17.73 ± 11.22	18.51 ± 10.55	22.19 ± 8.67	18.51 ± 10.88
	ROM (°)	36.84 ± 18.81	60.95 ± 9.97	56.27 ± 20.31	73.49 ± 14.83	54.23 ± 14.23	82.70 ± 10.55
Front ankle	Peak DF (°)	15.98 ± 4.19	10.37 ± 5.12	21.01 ± 3.16	11.95 ± 5.12	23.68 ± 4.19	16.55 ± 5.24
	Peak PF (°)	5.37 ± 3.99	−7.64 ± 7.23	9.56 ± 3.07	−9.69 ± 6.74	8.39 ± 5.96	−2.67 ± 5.65
	ROM (°)	10.61 ± 4.73	18.01 ± 6.34	11.45 ± 4.49	21.64 ± 4.68	15.29 ± 4.68	19.23 ± 5.31

Values are shown for each target distance condition (short, normal, long) under LWOA and LWA.

**Table 2:** Summary of two-factor ANOVA results for movement type (LWOA vs. LWA), target distance (short, normal, long), and their interaction on CoM peak velocity and lower limb joint angles.

Variable		Target distance condition								
		Distance (linear)F	*p*	*η*p²	Type (linear)F	*p*	*η*p²	Dist × Type (linear) F	*p*	*η*p²
CoM	Peak velocity (m/s)	566.35	*p* < .001	0.98	211.38	*p* < .001	0.95	26.47	*p* < .001	0.71
Rear hip	Peak flex (°)	70.74	*p* < .001	0.87	27.60	*p* < .001	0.72	21.31	*p* < .001	0.66
	Peak Ext (°)	75.08	*p* < .001	0.87	35.48	*p* < .001	0.76	61.47	*p* < .001	0.85
	ROM (°)	164.53	*p* < .001	0.94	92.39	*p* < .001	0.89	4.55	*p* = .056	0.29
Rear knee	Peak flex (°)	121.39	*p* < .001	0.92	16.42	*p* = .002	0.60	10.97	*p* = .007	0.50
	Peak Ext (°)	85.60	*p* < .001	0.89	30.15	*p* < .001	0.73	47.87	*p* < .001	0.81
	ROM (°)	239.63	*p* < .001	0.96	121.36	*p* < .001	0.92	24.96	*p* < .001	0.69
Rear ankle	Peak DF (°)	0.49	*p* = .499	0.04	20.44	*p* < .001	0.65	2.03	*p* = .182	0.16
	Peak PF (°)	120.73	*p* < .001	0.92	40.53	*p* < .001	0.79	42.20	*p* < .001	0.79
	ROM (°)	123.38	*p* < .001	0.92	0.23	*p* = .638	0.02	51.33	*p* < .001	0.82
Front hip	Peak flex (°)	140.07	*p* < .001	0.93	35.33	*p* < .001	0.76	18.98	*p* = .001	0.63
	Peak Ext (°)	58.51	*p* < .001	0.84	59.20	*p* < .001	0.84	3.68	*p* = .081	0.25
	ROM (°)	92.11	*p* < .001	0.89	2.09	*p* = .176	0.16	14.85	*p* = .003	0.57
Front knee	Peak flex (°)	301.75	*p* < .001	0.97	27.19	*p* < .001	0.71	0.42	*p* = .532	0.04
	Peak Ext (°)	1.13	*p* = .310	0.93	0.61	*p* = .450	0.53	0.42	*p* = .528	0.04
	ROM (°)	46.27	*p* < .001	0.81	24.23	*p* < .001	0.69	1.10	*p* = .316	0.09
Front ankle	Peak DF (°)	28.49	*p* < .001	0.73	61.92	*p* < .001	0.85	0.78	*p* = .396	0.07
	Peak PF (°)	20.22	*p* < .001	0.65	87.04	*p* < .001	0.89	0.31	*p* = .588	0.28
	ROM (°)	4.35	*p* = .061	0.28	53.83	*p* < .001	0.83	0.95	*p* = .351	0.08

For each variable, F-values, *p*-values, and partial *η*p² are reported for the main effects of Distance and Type and the Distance × Type interaction.

For peak CoM peak velocity, a significant main effect of target distance was observed (F = 566.35, *p* < .001, *η*p² = 0.98; Table 2). In LWOA, mean peak velocity increased from 1.02 ± 0.20 m/s (Short) to 1.81 ± 0.22 m/s (Normal) and 2.42 ± 0.24 m/s (Long). In LWA, values increased from 1.98 ± 0.19 m/s (Short) to 2.42 ± 0.21 m/s (Normal) and 2.88 ± 0.24 m/s (Long). A significant main effect of movement type was also detected (F = 211.38, *p* < .001, *η*p² = 0.95), with LWA exceeding LWOA across all distances. Importantly, a significant Type × Distance interaction (F = 26.47, *p* < .001, *η*p² = 0.71) indicated that the velocity difference between LWA and LWOA decreased from 0.96 m/s at Short distance to 0.46 m/s at Long distance.

For the rear leg, peak flexion and extension angles at the hip and knee joints, together with rear knee ROM and rear ankle peak plantarflexion, showed significant main effects of Distance and movement Type, as well as significant linear Type × Distance interactions (Table 2). Joint angle values increased progressively across target distances (Short < Normal < Long), and LWA exhibited higher values than LWOA. The significant interaction further indicated that the difference between movement types was greatest at Short distance and progressively diminished as target distance increased. As representative examples, rear knee peak flexion increased from 51.90 ± 9.63° at Short to 60.96 ± 5.25° at Long in LWOA, and from 56.08 ± 5.10° to 74.45 ± 6.12° in LWA (Distance: F = 121.39, *p* < .001, *η*p² = 0.92; Type: F = 16.42, *p* = .002, *η*p² = 0.60; Type × Distance: F = 10.97, *p* = .007, *η*p² = 0.50). Rear knee peak extension showed a similarly strong pattern (Distance: F = 85.60, *p* < .001, *η*p² = 0.89; Type: F = 30.15, *p* < .001, *η*p² = 0.73; Type × Distance: F = 47.87, *p* < .001, *η*p² = 0.81), with comparable effects observed for rear hip flexion, rear hip extension, rear knee ROM, and rear ankle peak plantarflexion (Table 2).

In contrast to the rear leg, front-leg variables showed significant main effects of Distance across several joint angles, with values increasing progressively from Short to Long target distances (Table 2). For example, front hip peak flexion increased from 36.08 ± 10.52° at Short to 80.43 ± 13.20° at Long in LWOA and from 59.48 ± 14.62° to 83.25 ± 14.50° in LWA (Distance: F = 140.07, *p* < .001, *η*p² = 0.93). A significant main effect of movement Type was also observed for this variable (F = 35.33, *p* < .001, *η*p² = 0.76), with higher values in LWA than in LWOA. However, Type × Distance interactions were not evident for most front leg joint variables. For instance, front knee peak flexion showed no significant interaction (F = 0.42, *p* = .532, *η*p² = 0.04), despite significant main effects of Distance and Type.

## 4 Discussion

We hypothesized that peak horizontal CoM velocity and key lower limb joint variables would increase with target distance in both movement types. In addition, we expected that LWA would produce larger absolute values and greater distance-related increases than LWOA. The present findings partially supported these hypotheses. Peak CoM velocity and key lower limb joint angle variables increased with target distance in both LWOA and LWA. LWA produced larger absolute values than LWOA across distance conditions. However, LWA did not consistently demonstrate greater distance-related increases, as the difference between LWA and LWOA decreased at longer target distances.

For CoM peak velocity, distance produced a strong main effect (*η*p² = 0.98), with values increasing from 1.02 ± 0.20 m/s at Short to 2.42 ± 0.24 m/s at Long in LWOA, and from 1.98 ± 0.19 m/s to 2.88 ± 0.24 m/s in LWA. Importantly, the interaction effect (*η*p² = 0.71) demonstrated that the velocity advantage of LWA decreased from 0.96 m/s at Short distance to 0.46 m/s at Long distance.

Focusing on the rear leg, peak flexion and extension angles at the hip and knee joints, together with rear knee ROM and rear ankle peak plantarflexion, increased with target distance in both LWOA and LWA. Moreover, all of these variables exhibited higher values in LWA than in LWOA. These findings are consistent with prior work (6), which reported progressive increases in rear knee flexion, rear hip and knee extension, and rear ankle plantarflexion as target distance increased. In addition, the present results corroborate earlier findings (4) demonstrating that rear hip peak flexion contributes to acceleration during the advance phase. Importantly, the distance-related increases in rear hip and knee joint angles, rear knee ROM, and rear ankle peak plantarflexion differed between LWOA and LWA. For example, in rear knee peak flexion, the difference between LWA and LWOA was 4.18° at Short distance and 13.49° at Long distance, while the increase from Short to Long was 9.06° in LWOA and 18.37° in LWA. Thus, the magnitude of change across distances was not identical between movement types. Notably, this interaction pattern paralleled the changes observed in peak CoM velocity, where the LWA–LWOA difference decreased from 0.96 m/s at Short distance to 0.46 m/s at Long distance (Table 2). Taken together, the concurrent modulation of rear-leg kinematics and CoM velocity indicates a close association between rear knee–hip motion and distance-dependent velocity control. This interpretation aligns with previous studies that identified rear knee and hip joint motion as key determinants of lunge velocity (2–4). Interestingly, although we expected that combining an advance with a longer lunge (Long LWA) would continue to augment CoM velocity and joint excursions, the gains were not as large as anticipated. This suggests there may be an optimal range of joint motion for maximizing lunge performance in LWA. Pushing beyond that optimal range (e.g., even deeper knee flexion or longer lunge distance) might not yield proportional increases in velocity. In fact, previous research in analogous movements has shown diminishing returns beyond certain joint angle optima. For example, in vertical jumping, large increases in knee flexion depth result in only minor improvements in jump height (16). Similarly, our findings imply that each joint has an optimal range for contributing to forward propulsion in the lunge. Once a fencer approaches those optimal angles, further increasing the range of motion is unlikely to produce additional CoM velocity gains.

For the front leg, previous research has reported significant differences between LWA and LWOA in hip and ankle joint angles and range of motion, indicating that the presence of an advance step influences front leg kinematic characteristics (4). In the present study, when target distance was included as a factor, several front leg joint angle variables and ROM values changed significantly with increasing distance. Both movement types shared a similar distance-dependent increase in these variables, while LWA generally exhibited higher peak angles and ROM values than LWOA. However, no significant Distance × Type interaction was observed for most front leg variables. These findings suggest that the distance-dependent change in velocity difference between LWA and LWOA is more strongly associated with changes in the rear leg rather than the front leg.

From a practical perspective, the present findings highlight the importance of distance perception and movement selection in fencing. The results suggest that attempting attacks from unnecessarily long distances may not proportionally increase peak velocity, even when greater joint excursions are observed. Accordingly, coaching should not simply emphasize attacking “from farther away” or “with larger movements,” but rather promote an understanding of which movement type is appropriate for a given tactical distance. For example, when the opponent is positioned at a Short-to-Normal distance, LWA may be advantageous because sufficient rear leg joint excursion and peak angles can be achieved, resulting in a clear increase in peak velocity. In contrast, as target distance increases, the velocity difference between LWA and LWOA diminishes, and the additional velocity benefit of the advance becomes relatively smaller. Therefore, at longer distances, LWA should not be selected solely to increase velocity; instead, movement choice should be made strategically, considering timing and counterattack risk, and incorporating appropriate distance management even when LWA is used.

This study has several limitations. First, the experimental conditions were restricted to three target distances and two movement types in a controlled laboratory setting. In actual bouts, opponent behavior, feints, and tactical dynamics may influence attack execution. Therefore, the present findings should be interpreted within the context of isolated lunge movements performed under predefined distances, and further research is required to extend these results to dynamic bout environments. Second, the present analysis was limited to kinematic variables and did not include kinetic data, muscle activation measures, or trial-level performance outcomes such as touch success or scoring indices. Consequently, we were unable to model the relationship between distance-, movement type–, and kinematic-related changes and performance outcomes using mixed-effects approaches (e.g., LMM/GLMM). Future studies incorporating kinetic measurements, electromyography, and performance endpoints across both successful and unsuccessful trials would enable a more comprehensive understanding of how movement strategies relate to performance. Third, the sample consisted solely of skilled male collegiate fencers. The results may differ in female athletes or fencers at different competitive levels; therefore, additional investigations including diverse populations are warranted.

## 5 Conclusion

This study examined changes in lower limb joint motion and peak velocity of the CoM during the LWA and LWOA in fencing under different target distance conditions. Results revealed that as the target distance increased, both actions exhibited significant changes in the horizontal peak velocity of the CoM and several joint angle variables. Furthermore, these changes were significantly larger in the LWA compared to the LWOA, and the magnitude of these changes differed between the LWA and LWOA. These results suggest that the rear leg primarily contributes to increasing the overall velocity of the attack action by generating propulsive force. These findings provide scientific evidence to support lunge strategy selection according to movement type and target distance, thereby contributing to performance enhancement in elite fencing.

## Data Availability

The raw data supporting the conclusions of this article will be made available by the authors, without undue reservation.
