# Supplementary figures and images for: Interaction of target distance and movement type on lower limb kinematics in fencing
Source: Front Sports Act Living. 2026 Mar 23;8:1756804. doi: 10.3389/fspor.2026.1756804 (PMC13050851; doi:10.3389/fspor.2026.1756804)

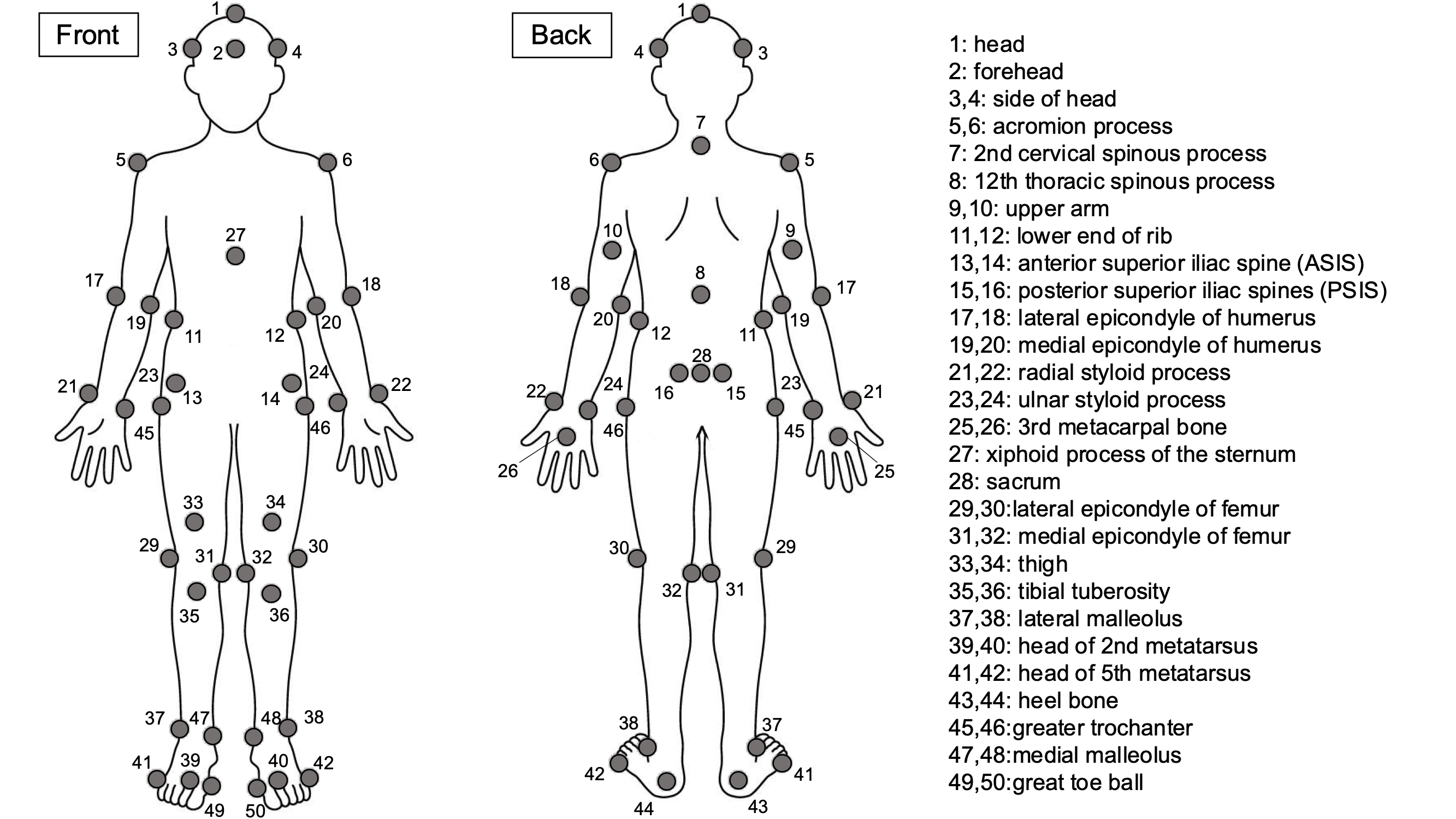

Supplement: Supplementary file 1 [file Image1.tiff]
